# Principes généraux de Physiologie-Pathologique, coordonnés d'après la Doctrine de M. Broussais

**Published:** 1821-06

**Authors:** 


### CRITICAL ANALYSES

OF

### RECENT PUBLICATIONS, IN THE DIFFERENT BRANCHES OF MEDICINE AND SURGERY,

### In the Literature of Foreign Nations.

Πατρίδες ἄρα
Ανδράσιν, ὦ πατραῖς ἄνδρες, ἀγαλλόμεθα.

*Principes géneraux de Physiologie-Pathologique, coordonnés d'après la Doctrine de M. Broussais.* Par L. J. Bégin. 8vo. pp. 390. Mequignon-Marvis, à Paris, 1821.

[In continuation from page 432.]

WE have passed over the preceding chapter more rapidly than we should have done, probably, had we not been desirous to devote the extent of space we have yet to appropriate to our account of this work, to that on *the general treatment of irritations;* and we enter on this subject with the more zeal, from a considerable proportion of English medical men—judging from the works of the older writers, or the incorrect accounts of superficial observers, ignorant of the principles which direct their conduct,—having erroneous notions of the merit of the practice of the French physicians, which they are pleased to regard with ridicule or contempt. Let us see whether Mr. Bégin's account of the therapeutic principles of the more enlightened part of them does not present matters which might be considered, more advantageously, with somewhat different sentiments.

The first questions which present themselves when we endeavour to determine what should be the conduct of the physician in the treatment of diseases, are these:—Is it, in general, more advantageous than injurious to the patient to leave to nature the care of his cure? And, if this conduct be favourable in certain cases, and the contrary in others, what are the diseases which the physician should always combat; and in what circumstances should he abandon them to their natural course?

The partizans of the "*medécine expectante*" have adopted the principle that, in acute diseases, nature is disposed to a regular course, the issue of which is almost always more advantageous, in its results, than that of the violent methods of those physicians who would take into their hands the direction of the progress of the malady. Apoplexies, intermittent fevers of a very bad type, and some other very severe affections, have alone appeared to them to be beyond the restorative powers inherent with living bodies. Chronic diseases, on the contrary, although submitted to the same regulative principle of the organic movements, have almost always been considered to be above the efforts of this power; and it has been said that they, most frequently, claim the active interference of the physician.

It has been observed, by those same persons, that the termination of violent disturbances of the functions is often accompanied with a sudden and intemperate re-establishment of the secretions, and the evacuation of a more or less considerable quantity of fluids. They have given to this remarkable phenomenon the name of crisis; and, generalizing the cases in which they have observed these crises to be followed by the prompt restoration of health, they have pretended that the physician should attempt to do no more than to combat symptoms of too great violence, to prepare and favour, or give rise to, the critical movements.

The suspension of the secretions, the burning heat and dryness of the skin, the thirst which torments the patient, the smallness in quantity and turbid state of the urine, are, in acute fevers, so many phenomena dependant on the irritation which is the source of the general disease. If this primary irritation suddenly ceases, either by the spontaneous actions of the economy, or by the effects of certain medicines, it is a natural consequence that the secondary phenomena which they have produced should cease at the same time. We observe then that the secretions are suddenly re-established, and with a degree of energy more considerable in proportion as they have been more completely arrested, and as the organs which are charged with their elaboration have been in a state of more intense sympathetic irritation. If we examine the patient at the time the crisis is about to take place, we find that the irritation of the organ primarily affected is becoming less severe, and that there is substituted an excitement of the secretory organs, the intensity of which increases in proportion as the other diminishes. It seems probable, then, that it is not because the "*crisis*" appears that the malady is alleviated; it is because the irritation of the parts primarily affected has subsided, that the secretions are re-established: the critical phenomena are the results, and not the causes, of the cessation of the disturbance excited by the inflammation of the viscera.

These different views have given rise to two different modes of medical treatment: that in which the critical efforts are regarded as the cause of the disappearance of the primary disease, has led to the adoption of the "*medecine expectante;*" that in which they are considered as results of its cessation, to the practice which will be described after we have shown somewhat more particularly the indications to which it is referable.

Many phenomena which authors have not considered as appertaining to crises, should however be allied with them, since they depend on the same vital laws, and lead to analogous effects, though the final results are sometimes different. We have seen, for example, that, when an organ is irritated, the parts more intimately related with it by sympathy participate with its irritation. Now it happens, very often, that this secondary irritation substitutes the primary, and thus terminates the original disease. It is in this way that cutaneous eruptions, boils, erysipelas, and many other affections, which succeed to gastritis, coincide with the disappearance of the latter disease after having been excited by it. It is by the same laws, however, that other diseases originate which are sometimes more severe and dangerous than the original one. Thus, in children, the brain, sympathetically excited by irritation of the stomach and intestines, becomes in a few days the principal seat of disease, which will probably be more readily fatal than the gastric disorder. Sometimes the primary disease accompanies the secondary affection; and, in this case, the latter frequently seems to aggravate, rather than to alleviate, the former.

It has been generally observed, that the organ which predominates in the economy, and which characterizes the special idiosyncracy of the subject, becomes almost always the seat of the secondary diseases which substitute the primary; or any other part which has been frequently much excited, and thus become the most irritable point in the body, will become the seat of the substituted affection. The physician may, then, in a great number of cases, be able to determine the organ in which the secondary disorder will appear.

Substitutions of irritation analogous to those we have seen take place from the internal organs to the external parts, will also be effected between the latter and the former. This phenomenon, although it occurs in an inverse direction to that above considered, is nevertheless a consequence of the same principle, and is effected by the same mechanism. It is thus that, on the skin being the seat of irritation, this affection may disappear suddenly, and the mucous membranes of the stomach and intestines, or of the lungs, will become the seat of more or less violent irritation. A man is attacked with irritation in one or more of his joints: the digestive organs are then sympathetically affected; the pain in this seat is soon, or immediately, followed by the cessation of the primary disease, and a gastro-enteritis succeeds to it. It is not then said that gastritis is the crisis of gout, and yet, for the sake of congruity, it should be said; since, in the case where gout succeeds to gastritis, it is pretended that the affection of the joints is the crisis of that of the stomach. In many instances, cutaneous diseases provoke internal inflammations, and disappear only on leaving behind them very serious maladies. Here the spontaneous efforts of the economy, far from being favourable to the patient, are evidently the contrary, and these effects of the *vis medicatrix naturæ* are even frequently fatal.

An important fact to be observed in the history of crises, is that, as the irritation is more acute, so is its disappearance more rapid: the more vigorous the patient, too, the more apparent are the critical phenomena, and generally more prompt in producing their proper results, whether they be for good or evil.

When irritation is developed, it is certain that, in submission to the organic movements, it tends to a termination after a longer or shorter period. Now, this termination may happen either only after a very long and almost indefinite period, or by the death of the patient, or with the re-establishment of health, which is operated with or without a *crisis.* Let the patient be cured, that is the object of the physician; and, in order that the medical art may be as beneficial as it is qualified to be, let the disease be removed as promptly as possible. Whilst it exists, the patient suffers, and he is submitted to all the hazards which accompany its presence, and the possibility of its exasperation. It is perfectly indifferent to the individual who is cured of a severe disease, whether he has had a *crisis* or not. Besides this, the expected crisis, the precursors of which are watched with so much solicitude, is often wanting, when the disease is left to the spontaneous efforts of the system, or it is operated by a secondary irritation which is more dangerous than the primary; and, whilst we are observing the progress of the disease through its long course, unforeseen accidents may destroy the patient, or the irritation may become more obstinate and pass into a chronic form; or a secondary affection arises without the disappearance of the primary, and then we have two diseases to treat instead of one.

The foregoing remarks, (although they are but slight hints of what might be said on this subject), with the consideration that it is in the power of medicine to effect the desired object, seem to establish the axiom of the propriety of combatting irritations wherever they may be seated. This point being settled, it remains for us to consider the methods which are the most appropriate for the treatment of the diseases under consideration. The methods generally adopted consist in —1°. The application of general antiphlogistic measures and local blood-letting; 2°. The application of irritants to parts remote from the seat of the disease, in order to elicit the irritation from the latter; 3°. The topical application of stimulants to the part itself which is the seat of irritation. The second of these methods is founded on indications derived from the views of the spontaneous efforts of the economy that were above exposed: but let us examine each of the three in a particular manner, in order to determine its relative value to the rest, and the cases to which it is most properly applicable.

The first is, in general, the most efficacious, and that which is attended with the least inconvenience to the patient; and it is that which should be employed, for the most part, in the early stages of irritations. It must not, however, be supposed that it may be applied without discrimination, or without some modifications on particular occasions. When the irritation is very intense, and the pain attending it suspends, almost completely, the influence of the nervous system on other organs; and the heart, hardly able to contract, propels but a very small quantity of blood into the arteries, it would be imprudent to effect at once copious sanguineous depletion: such depletions have, not unfrequently, produced immediate death. The physician should then prescribe the application of leeches, and these in some instances should be only few in number: he will observe their effects, and, if the pulse become developed, he will then revert to this means in a less restricted manner. It is not a rare thing to see patients, thus roused from the most profound oppression and extreme debility, present symptoms of excitement sufficiently violent to indicate general blood-letting; whilst, a few hours previously, they would, probably, have borne hardly a dozen leeches. In the contrary case, that is to say, when local blood-letting, although moderate in extent, instead of leading to an increase of fulness and strength of the pulse, is followed by contrary circumstances, and the debility is increased, the indication is evident: sanguineous evacuations must then be abandoned, and revulsives, that is, counter-irritants, should be resorted to, in order to elicit to the external parts the vital actions concentrated in the internal organs.

In subjects which present the phenomena of excitement of the sanguineous system to a high degree, it is easy to determine the conduct which it is proper to adopt: general and local blood-lettings, spare diet, and cool drinks, are indispensable, and their salutary effect is assured. It sometimes happens that a state of extreme plethora fetters the organic movements, and produces a state of debility that is merely apparent: some little acuteness is often necessary in order to discern this condition; and it requires, more imperiously than the preceding one, abundant emissions of blood, and the antiphlogistic diet to the most rigorous extent. The subjects of this condition present, generally, a very large chest, a deep-red colour of the surface of the body, especially in the face; the features of the countenance are unusually large; and there is a certain embarrassment in their voice and respiration which indicates that the chest is surcharged with blood.

In cases where irritations occur in subjects previously shaken by disease, exhausted by previous excitement, and emaciated by chagrin or a penurious and unwholesome diet, the state of the patient is often sufficiently embarrassing to perplex the most sagacious practitioner. We must take care here not to evacuate, by general blood-lettings, the small quantity of blood which is present in the external veins. By depriving remote parts of the fluid which excites them, we should augment the relative predominance of the irritated organ; for it would participate but very little in the effects of the depletion. Concentration of the vital actions is, moreover, operated with much greater facility in enfeebled subjects than in others; and it is more difficult to destroy their irritations by remote bleedings, because the irritated organs retain their undue proportion of blood with more tenacity. It is necessary, then, to apply our remedies as nearly as possible to the seat of the disease: it would even be desirable that we should immediately, without augmenting the irritation, subtract blood from the part affected; but this is not in our power in affections of the internal organs, and we must be content with operating on the part of the capillary system that is most intimately allied with them.

In cases of inflammation of the mucous membranes, it is the capillary system of the skin which is most intimately related with the parts in question, and it is, consequently, on this part that leeches should be applied; but, in acting thus, we might deprive the system of so considerable a quantity of blood, in regard to the condition of the patient, that death ensues as a consequence of it. When, then, the relief of the local disease is not equivalent to the general debility produced by the blood-letting, the effect of this measure is disadvantageous. In such cases, it is more proper to have recourse to irritants on exterior and remote parts.

The propriety of blood-letting, in general, is here the more insisted on, because the blood, although it is elicited to the part effected chiefly by the excitement of the nerves and vessels of this part, is itself a powerful cause of excitement, which it is desirable to obviate. We know that the blood is more abundant in a given part as the vitality of this part is more active; and that, on compressing the arteries running to it, without totally arresting the circulation, we weaken the sensibility with which it is endowed; or, in other words, by diminishing the quantity of blood which flows through a certain texture, we render the vital actions of this texture less energetic. If we prevent, for some time, the afflux of blood to an irritated organ, the excitement of it ceases. It is thus that compression, methodically applied, opposes the inflammation of parts after strains, and removes inflammation of structures that can be duly submitted to its influence.

There are persons in whom irritations are so obstinate, and so tenaciously fixed in the parts affected, that it is almost impossible to destroy the morbid action, although we employ general and local blood-lettings, emollient applications, and the antiphlogistic regimen, to the utmost extent. Indeed, in proportion as we go on practising blood-letting in such cases, the patient becomes enfeebled, whilst the irritation seems to become more and more obstinate: sanguineous evacuations would produce death rather than the removal of the inflammation. If we persist in the use of them, the patient becomes emaciated, sinks with astonishing rapidity, and arrives at a state of extreme marasmus, without the inflammation having been dissipated. It is advisable, as soon as we perceive this disposition in the case, to refrain from further depletions to much extent. The first period of the disease, that in which it is possible to remove suddenly the irritation, being passed, the disease will run its course, and the functions will be for a long time disturbed. The strength of the patient should here be carefully managed; we should gain nothing by depressing it too much or too rapidly: we must give revulsive measures with the administration of sedatives internally, and have recourse to the application of leeches occasionally, as the intensity of the symptoms may render them necessary.

A condition necessary in all cases, in order that irritations may be successfully treated, is the absolute rest of the irritated part. Our physiology shows us that the exercise of the functions is a powerful cause of stimulation for the organs which execute them: the actions performed by a given part cannot take place without the blood being elicited into its capillary system, and the nervous powers being excited. It is incontestible that inflammation must be exasperated by a cause which is followed by such results. Irritations of the internal organs, the progress of which is the most rapid, and which lead to the most serious consequences, especially require a state of repose of the affected organs. It is hence that silence and inaction of the body are so necessary for the cure of pneumonia and pleurisy. It may be remarked that general bleedings act beneficially in the former of these diseases, not only by depletion of the vessels, but also by diminishing the mass of fluid the lungs have to elaborate; and thus they favour the repose we endeavour to give to those organs: and if very violent inflammation of the lungs constitutes, frequently, so obstinate a disease that it is not possible to remove it suddenly by means of blood-letting, as some other inflammations may, it depends, probably, on our not being able to effect a complete suspension of the functions of the organ, the exercise of which constantly tends to preserve the excitement of its tissue. In irritations of the digestive organs, we can, by withholding the administration of alimentary substances, produce a suspension of their proper functions; and the success of our treatment will commonly be in proportion to the degree of strictness with which this privation of food is insisted on.

The second method, or that constituted of revulsive measures, may often be advantageously joined with the direct antiphlogistic means, when abstraction of blood has not been at once successful; but it is necessary to select with caution the period at which these measures are employed. If they are resorted to in a precipitate manner, they may give new activity to the original irritation, augment, in cases of gastro-enteritis, the arid heat of the skin, the frequency of the pulse, the dryness of the tongue, and the thirst, and render necessary further sanguineous evacuations.

It will be generally found that, whenever a remote irritation does not cause a cessation of the primary irritation, it increases, sympathetically, the violence of the latter; or, in the words of an English author, whose therapeutical disquisition constitutes one of the most interesting and valuable productions in medical literature, the irritation we produce in this case, instead of imitating the substituted, exemplifies the extended, disease. So that, whenever we judge it proper to have recourse to external irritants, we should use them in an energetic manner, and let their application coincide with that of antiphlogistic means; so that sedatives and refrigerants may be applied to the organ suffering the irritation, at the same time that the counter-irritants are placed on remote parts. By acting thus we may avoid the evils which are otherwise hazarded by the use of means of the revulsive kind.

The skin is the organ to which counter-irritants are most commonly applied: its exquisite sensibility, and the little danger attendant on irritations of it to a moderate extent, are the chief reasons for this choice; but it is not the only tissue on which those remedies may be successfully applied. They may, indeed, be administered internally, in cases where the intestinal canal is not the seat of irritation; and, when thus employed, they act beneficially in proportion to the sensibility of the mucous tissue on which they are placed.

Although it is almost indifferent what species of stimulant be applied externally,—the principal object of consideration being the diversity of the energy of the substance employed,—it is of the highest importance that a judicious choice should be made in the substances with which we irritate the intestinal tube. The membrane which lines this organ possesses a sensibility which develops such very different, and frequently opposite, results, according to diversities in the matters placed in contact with it, that the most dissimilar consequences often ensue from stimulation effected by bitter, aromatic, alcoholic, and purgative, substances. Another circumstance which should contribute to render the practitioner careful in his selection of means of this kind, is this, that the substances employed may be absorbed and carried into the general circulation of the fluids, and thus cause various irritations in the system, more or less generally. Those substances should ordinarily be preferred that irritate the intestinal tube without acting with violence on the sanguineous system: amongst the matters of this class, purgatives hold the first rank. Besides the irritation they oppose to that we wish to remove, they excite, in the mucous membrane of the intestines, a very considerable organic movement, the object of which is the secretion of a greater or less quantity of mucous matter. This process requires the afflux of an inordinate quantity of blood; it sets into vigorous action the nervous faculties of the abdominal organs; and, when it is entertained for a considerable period, it is but rarely that nature does not abandon, sooner or later, the morbid operations it was carrying on in remote parts. There is also another advantage derived from the use of purgatives, when judiciously employed, which is the separation of a more or less considerable quantity of the fluids from the general mass; an effect which produces a degree of debility often equally beneficial with that which follows evacuations by blood-letting, whilst its duration is less permanent. Some choice is necessary in the species of purgatives employed; the milder sort being generally preferable for patients of the sanguineous temperament; whilst the more active, and even drastic, are often most beneficial in persons of an indolent or phlegmatic constitution. Bitter medicines are preferable to purgatives in scrofulous subjects, and during chronic and indolent inflammations of the lymphatic system. Besides the irritative action they exert on the mucous membrane, they excite the sanguineous system, and restore to it the predominance it should preserve over the lymphatic vessels. We obtain here, then, a local revulsive effect, by the irritation of the mucous membrane of the stomach and small intestines; and a general revulsive effect, which consists in eliciting the vital actions to another order of vessels than that which is the seat of disease.

In addition to what has been said respecting the use of counter-irritants on the skin, it should be remarked that it is not a matter of indifference whether they are placed near to, or remote from, the part which is the seat of the disease. Whilst the irritation is very acute, we should apply the irritating topics to distant parts: it is in conformity with this precept that vesicatories are successfully placed on the lower extremities in acute gastro-enteritis; the feet, knees, and even the elbows, may be especially surrounded with rubefacient sinapisms, or vesicatories, in this case. Irritations about the large joints is preferred, because these parts are known to be related with the mucous membranes of the digestive organs by a particular sympathy, and that excitement of them exerts a more direct and powerful effect on those organs than excitement of most other parts. In proportion as the irritation has been prolonged, it is advantageous to approach the counter-irritation towards the seat of the former affection; and, when this is of a chronic kind, the use of the remedial measure should not be suspended until the disease has entirely disappeared.

The revulsive measures are not constituted solely of the use of topics which excite irritation of surfaces; there are various other measures which produce somewhat analogous consequences. Thus, exercise of the limbs, in the greater part of chronic inflammations of the viscera, is a resource as simple as it is efficacious, and one which should never be neglected. Muscular exertions elicit the vital movements to the exterior parts of the body; and they produce a revulsive action which may be lessened or increased any instant, according to the existing indications. Their energy is very different from that of a blister, to which the economy becomes insensible after it has existed for a few days. During convalescence from chronic diseases of the viscera, this measure is almost indispensable, and is the most powerful means of obviating a relapse. The extreme rarity of deaths amongst soldiers,—even when by no means free from sickness in their ranks,—during long and difficult marches, is a fact which has been remarked in the most general manner.

The third method for the treatment of irritations,—that in which irritating substances are applied to the seat of the disease,—is, of the whole, the least advantageous; that which most readily leads to an exasperation of the malady, and which, consequently, submits the patient to the greatest number of unfavourable chances. The circumstances under which it may be convenient to employ it are, then, but rare; and well-cultivated experience and much sagacity are requisite in the practitioner, in order that they may be recognized. The cases to which it is applicable, are the following:—

1°. When the morbid irritation is intermittent, and when the intervals of the paroxysms are perfectly exempt from super-excitation. In this case, powerful stimulants, amongst which is the cinchona, are of the greatest efficacy; but the first and most important of the conditions on which the success of the treatment depends, is that the state of apyrexia be complete; and, in proportion as it is less perfect and of short duration, it becomes more difficult to effect the cure. When the excitement is sufficiently intense to keep up, constantly, a febrile commotion, but not sufficient to prevent a recurrence of rigor,—that is to say, when the fever is remittent,—the first indication is to alleviate the irritation; and if, after the diminution of this, the periodicity remains, leaving intervals of complete absence of fever, it will be proper to employ cinchona. The theory of the agency of this remedy is pointed out in former parts of this article.

2°. Another case in which stimulants applied to a part affected with irritation are frequently successful, is that of certain hemorrhages, and especially of hemorrhages seated externally. Stimulants seem to act here by causing the vessels to contract, or by temporarily rendering them torpid, after the first excitement from their application has subsided; but, when the vessels are intensely irritated, and when the patient is very vigorous, the re-action which follows their application often aggravates the accidents, and renders the hemorrhage more violent. Sometimes they seem to arrest the flow of blood only by substituting a real state of inflammation to the hemorrhagic irritation. Now, this conversion may take place when internal organs are the seat of the disease, and, such inflammation being often not less dangerous than the lesion which has preceded them, the prudent physician will not use these means internally without the most careful circumspection.

3°. When the secretory vessels are especially affected with irritation, we may, in certain cases, substitute for this irritation a state of real inflammation, the course of which will be shorter than that of the former condition, whilst it terminates in a more assured cure. But remedies of this kind, though accompanied with but little danger when they are applied to the urethra, the conjunctiva, and several other surfaces, are attended with serious hazard when the digestive tube is the seat of the disease. Violent irritation of the mucous membrane of the urethra, or of the eye, may be borne without much peril, whilst that of the stomach and intestines would soon be mortal. There is a close analogy between hemorrhages and too-abundant secretions, and therefore the indications for the use of remedies of the kind under consideration, are similar in both cases ; as is, also, the theory of their mode of agency.

Such is the outline our limits will permit us to trace of the clinical practice of the more enlightened part of the French physicians: it does not present a single trait that is not copied from the picture drawn by Mr. Bégin ; and those who may consider the representation it gives of its object importantly defective, (as an outline,) or devoid of keeping, are requested to refer to the original, and not to form their judgment before they have contemplated it when filled up and coloured by the hand of an able master.

---

